# Biodiesel production from palm olein: A sustainable bioresource for Nigeria

**DOI:** 10.1016/j.heliyon.2020.e03725

**Published:** 2020-04-14

**Authors:** Felix Ishola, Damola Adelekan, Angela Mamudu, Temitope Abodunrin, Abraham Aworinde, Obafemi Olatunji, Stephen Akinlabi

**Affiliations:** a Mechanical Engineering Department, Covenant University, Ota, Nigeria; b Chemical Engineering Department, Covenant University, Ota, Nigeria; c Physics Department, Covenant University, Ota, Nigeria; d Mechanical Engineering Science Department, University of Johannesburg, South Africa; e Mechanical Engineering Department, Walter Sisulu University, South Africa

**Keywords:** Energy, Bioconversion, Renewable energy resources, Energy sustainability, Fuel technology, Industrial energy consumption, Palm olein, Biodiesel, Cleaner energy, Transesterification, Policy

## Abstract

Dangerous environmental consequences and market unpredictability of fossil fuels have necessitated the need for sustainable large-scale production of biofuel in Nigeria. Unrefined palm oil (UPO) is a significant product of commercially available oil palm plants in the country. This study experimentally investigates the production of biodiesel from refined, bleached and deodorised (RBD) palm olein extracted from UPO obtained from batch reactors. The transesterification process of the RBD palm olein with methanol and in the presence of potassium hydroxide (KOH) catalyst produced biodiesel with a 62.5% yield, thus confirming its feasibility for mass production. The derived biodiesel has properties equivalent to ASTM D792 standard for biodiesel fuels.

## 1 Introduction

### 1.1 Background

Before the 19^th^ century, biomass was the leading source of energy the search for more efficient energy continues as technology advances. Dried wood was a predominant fuel for cooking and heating, while ethanol and vegetable oil was the primary fuel used for lighting. Early men also burned wood to warm shelters and for heat-treating clay artefacts (Guo et al., 2015). Over many centuries, industrialisation energy demands had engendered the transitions of energy regimes from wood to coal and from coal to fossil fuel, respectively (Mosarof et al., 2015).

Consequently, fossil fuels eventually gained dominance as a source of energy, recently covering about 48% of global energy demand (Emodi, 2016). The limitations of intensive usage of fossil fuels such as depleting global reserves, continuous environmental pollution and market unpredictability had justified the increasing research and development in fuel alternatives in all countries (Mamudu and Olukanmi, 2019; Naik et al., 2010; Olatunji et al., 2019a,b). Biofuel is an alternative fuel speedily gaining applications in the modern age. Biofuels are either raw or extracted products from plant biomass that can release their embedded energy (Guo et al., 2015). Amongst available biofuels, biodiesels are considered more sustainable, cleaner and fuel options (Thushari and Babel, 2018). Besides, applications of biodiesels in compression ignition (C.I.) engines give some satisfactory performances (Jambulingam et al., 2019) (Keera et al., 2018).

Sir Rudolph Diesel invented the first compression ignition (C.I) engine around the 1890s, and he operated it on extracted oil in raw form in 1900 at the World's Exhibition in Paris (Mata et al., 2010) (Zahan and Kano, 2018). The practice of using vegetable oil as automotive fuel continued up to 1920s when fossil fuel completely took over the function of powering vehicles (Guo et al., 2015). Fossil fuelled diesel engines were increasingly utilised in agricultural and mechanical engineering applications due to its higher capacity to produce energy in comparison to other engines (Kaisan et al., 2017) (Venkatesan et al., 2019). However, diesel engines significantly contribute to environmental pollutants based on its higher (i) combustion temperature, (ii) inappropriate fuel combustion and, (iii) sulphur and carbon contents (Gopidesi et al., 2018).

Biofuels are useful in C.I. engines as both stand-alone fuels and diesel blend (Mukhtar et al., 2019). The fact that biodiesel is a cleaner fuel usable in current diesel engines with/without engine alterations makes it a feasible option being actively researched (Gashaw and Getachew, 2015). The advantages of vegetable oil-based biodiesel over conventional diesel fuel include increased profitability, biodegradability and low aromatics and sulphur contents (Monisha et al., 2013). Its possession of higher flashpoint in comparison to petroleum-derived diesel makes it one of the safest and non-toxic fuels option (Aransiola et al., 2013). Chabisha P Makgaba and Daramola (2015) observed that the application of biodiesel within a diesel internal combustion system improved the lubricity of the system and extended the catalytic converters life when compared to the base system. The system production process and general utilisation cuts down emissions drastically to about seventy-eight percent when compared to petroleum-derived diesel. Its limitations are more significant NO_x_ discharges, lower calorific value, higher viscosity and density value (Devarajan et al., 2017).

Edible vegetable oils such as soybean oil, palm oil and sunflower oil are first-generation biodiesel feedstocks because they were the first type of crop used to produce biodiesel (Bowyer et al., 2018). Non-edible vegetable oils such as jatropha, mahua, jojoba oil, salmon oil and sea mango, animal fats, used cooking oils, agricultural and solid municipal wastes and are referred to as second-generation biodiesel feedstock (Aditiya et al., 2016; Olatunji et al., 2019a,b). The third-generation biodiesel feedstock is microalgae, cyanobacteria and other single-celled oleaginous microorganisms (Oyedepo et al., 2019). More recent research has produced fourth-generation biofuels feedstocks which are genetically engineered plants that consume more CO_2_ from the atmosphere than they may emit as fuel later during combustion (Shokravi, 2019). Fourth-generation technologies also include pyrolysis, gasification, refining, solar-to-fuel, conversion of vegetable oil and biodiesel to bioethanol and biogas using advanced technologies, as well as genetic manipulation of organisms intended to secrete hydrocarbons (Abdullah et al., 2019).

### 1.2 Palm olein as a sustainable local biofuel feedstock in Nigeria

Nigeria is struggling to get over her energy crises (Ekpe and Umoh, 2019) and at the same time, making efforts to promote low-level greenhouse gas (GHG) emissions (O. Olatunji et al., 2018). Nigeria as a country has made little progress in establishing sustainable energy system amidst many unimplemented policies (Ishola et al., 2019), and her biofuels industry is not an exception (Odetoye et al., 2019). Different types of biofuels emanate from different countries, depending on their available bioresources (Wu et al., 2019), and the technology considered most appropriate and economical (Avagyan and Singh, 2019). Experimentation and continuous research are enabling the development of viable biodiesel feedstocks and suitable production procedures (Abbaszaadeh et al., 2012). With vast arable land across Nigeria, the capacity for expanding its bioresources is feasible (Oyedepo et al., 2019). Direct comparative analysis has shown that the Nigerian oil palm industry has the prospect of producing biofuels commercially (Ishola et al., 2013).

Worldwide, palm trees account for around 10% of biodiesel production and are proliferating rapidly (Szulczyk and Khan, 2018). Compared to other oilseeds such as soybeans, rapeseed or sunflower, oil palm is the most efficient and cost-effective biodiesel feedstock measured in terms of oil yield per unit hectare of cultivation land area per annum (Ali et al., 2019; Lam et al., 2019). Also, palm oil has a higher yield of biodiesel production when compared to other bioresources (Ghazanfari et al., 2017). Table 1 indicates that Nigeria is the fifth-largest oil palm cultivator in the world. Taking references from other oil palm-based biodiesel producing countries like Indonesia and Malaysia (Silalahi et al., 2020), Nigeria stands a definite chance of having an established biodiesel market. Contrary to a consensus that palm oil as a feedstock for biodiesel will create food-energy nexus concerns (Olarinde and Olawuyi, 2013); availability of palm olein based on of consumer preference can be an economic advantage for commercial biodiesel production from palm olein. Traditionally, Nigerians prefer a high percentage of palm stearin based palm oil as culinary. Populace prefers to consume palm stearin compared to the palm olein fraction (Thomas et al., 2011).

**Table 1:** Recent World Oil Palm Production status (FAO, 2018).

Rank	Country	Production (Million Tonnes)
1	Indonesia	40.5
2	Malaysia	19.5
3	Thailand	2.8
4	Colombia	1.6
5	Nigeria	1.1
6	Guatemala	0.9
7	Honduras	0.7
8	Papua New Guinea	0.6
9	Ecuador	0.6
10	China (Including Mainland)	0.5
	Others∗	2.9
	Total	71.7

∗ Others includes Brazil, Cote d’Ivoire, Ghana, Cameroon, Costa Rica, Peru, Philippines, Congo, and few others with minimal contributions (Lam et al., 2019; Ishola et al., 2020b).

Many experimental works had earlier been conducted on converting palm kernel oil (PKO) to biodiesel within Nigeria (Akhabue and Ogogo, 2018; Aladetuyi et al., 2014; Alamu et al., 2007; Bello et al., 2015; Kareem et al., 2017; Tarbuka et al., 2017). However, there are scanty reports of biodiesel production from other oil palm products like palm olein. Palm olein can become a sustainable option for biodiesel production because it is a bye product of palm oil (Pannilawithana and Pathirana, 2017). Both unrefined palm oil (UPO) and palm kernel oil are primary oil sources from oil palm trees (Gourichon; 2013). In oil palm refineries, palm oil separates into palm stearin oil (which has a high-melting-point) from the low-melting-point product (that is, palm olein). The palm olein fraction has a higher number of long-chain and unsaturated fatty acids than the stearin oil (Cardoso et al., 2014). Palm olein is extracted from oil palm through three identified means. Firstly, as the less-dense upper layer product of refining UPO. Secondly, liquid fractionation processing of UPO produces a more refined palm olein referred to as RBD palm olein (See Mba et al., 2015). Also, palm olein can be obtained from liquid fractionation and crystallisation processes of palm kernel oil. However, it is sometimes considered as the right type of frying oil, but notwithstanding, an excellent feedstock for biofuel production (Lee and Ofori-Boateng, 2013).

**Figure 1:**
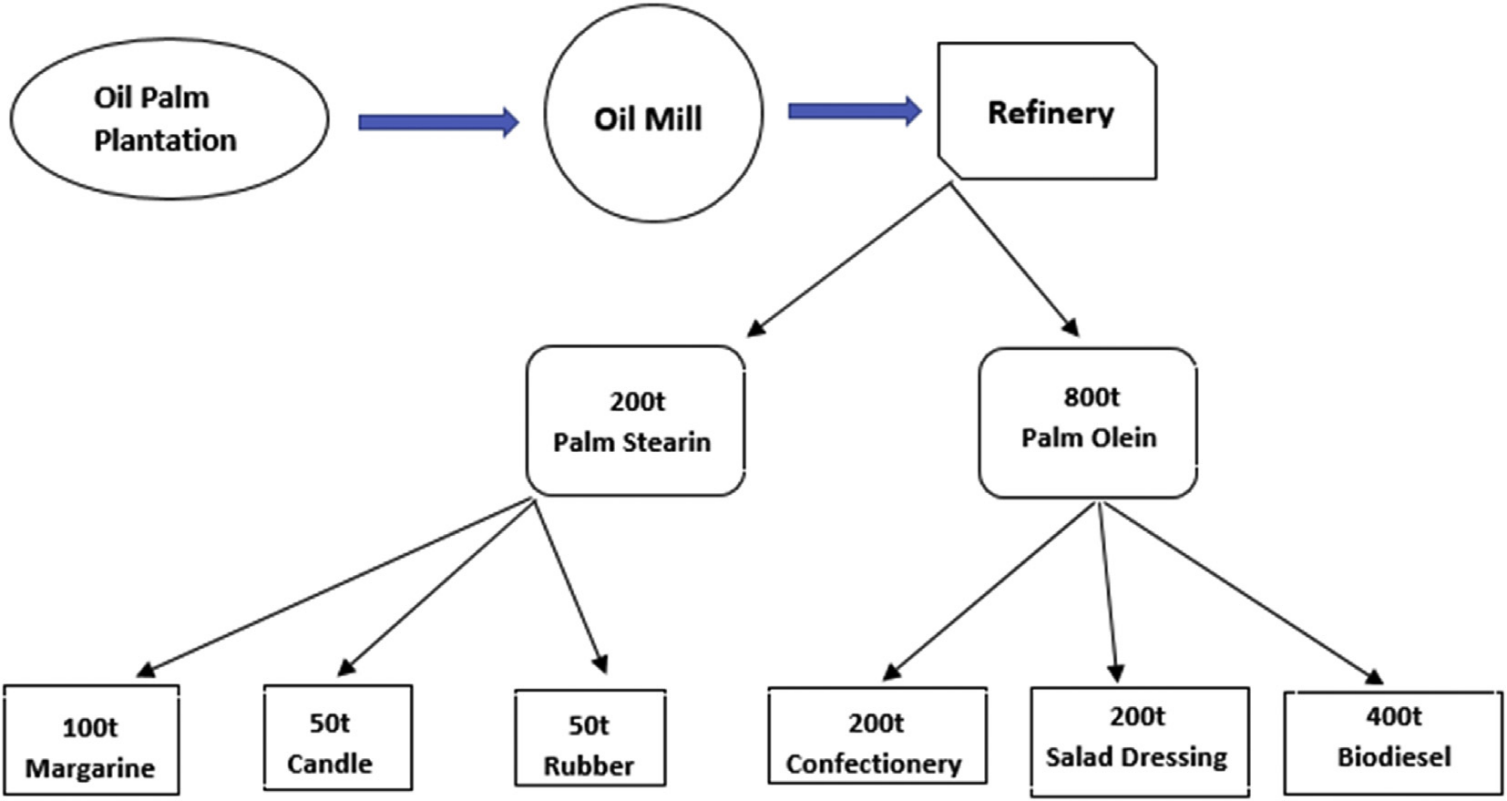
A simplified supply chain analysis for palm oil products in an Indonesian market model (Schleicher et al., 2019).

Generally, the final products of palm oil in a refinery includes palm stearin, palm olein and other derivatives of palm oil. They are readily used for three primary purposes: human consumption, animal nutrition and bioenergy (Mba et al., 2015). Figure 1 summarises a typical palm oil supply chain model, presented in Tons(t). The model was designed from an Indonesian palm oil downstream data observation system (Schleicher et al., 2019). According to Figure 1, a batch of crude palm oil mill produces 80% palm olein and 20% palm stearin. The model also affirms that about 50% of the palm olein ends up as processed biodiesel, while about 25% is processed for cooking purpose. Overall, about 40% of batch production from palm oil mills can be converted into biodiesels (Mba et al., 2015). Based on Nigerian consumers’ notion that palm oil with a high percentage palm stearin has many health benefits; we experimentally assessed palm olein as a locally available, clean and economical bioenergy source for biodiesel IC engines. On these leverages, investigating the feasibility of commercial-scale production of biodiesel with adequate yield from locally sourced Palm Olein is justified.

### 1.3 Biodiesel production process

There are a few alternative biodiesel production processes that exist. Some of these methods of producing biodiesel are here briefly discussed. The microemulsion is a stable thermal dispersion of immiscible liquids stabilised using surfactants or co-surfactants for biodiesel production (Monisha et al., 2013). Microemulsion method shortens production time for the biodiesel production (Gebremariam and Marchetti, 2017). The technique reduces the production costs, while the produced biofuel has increased cetane number. This is because there is no undesired reaction and by-products formation (Adepoju et al., 2018). However, the biofuels produced by emulsification and micro emulsification tends to yield more carbon and lacquer deposits on tips of injectors, intake valves and cylinder liners tops during usage in engines (Liaquat et al., 2013). These tendencies were linked to higher viscosity, lower volatility and reactivity of unsaturated hydrocarbon chains characteristics (Liaquat et al., 2014). This condition may eventually induce the sticking of the injection needle and incomplete combustion (Abed et al., 2019; Tziourtzioumis and Stamatelos, 2019; Pattnayak et al., 2019).

Thermal cracking is a conversion process that involves heating in the absence of oxygen or with the use of a catalyst (Abbaszaadeh et al., 2012; Tambun et al., 2017; Ishola et al., 2020a). The process is also known as pyrolysis. Studies revealed that thermal cracking of vegetable oil to biofuels yields alkanes, alkenes, alkadienes, aromatics and carboxylic acids in different proportions (Gashaw and Getachew, 2015). It is difficult to control the main product of pyrolysis because the process depends on experimental conditions like residence time of the heat, reaction temperature and heating rates (Girish, 2019). This phenomenon can lead to the formation of unwanted chemical compounds like sulphur, water and residues that induce engine corrosion (Jambulingam et al., 2019). Besides, adverse environmental reactions occur when vegetable oils possessing sulphur contents are combusted; it may result in SO_2_ emissions, which forms acidic rain (Demirbas, 2008). Thermal cracking is usually not considered for biodiesel production because it is an expensive process with higher maintenance costs incurred for the various fraction separations (Abbaszaadeh et al., 2012). Also, restrictions on final products and the overall difficulties associated with the processes across the various reaction paths and products make it unpopular for biodiesel production (Gashaw and Getachew, 2015). Among the mentioned methods, transesterification stands to be an adequate choice because of its process simplicity and the fact that fatty acid ester obtained by this process has proximate characteristics to that of diesel (Kapadia et al., 2019).

### 1.4 Transesterification as a biodiesel production process

**Figure 2:**
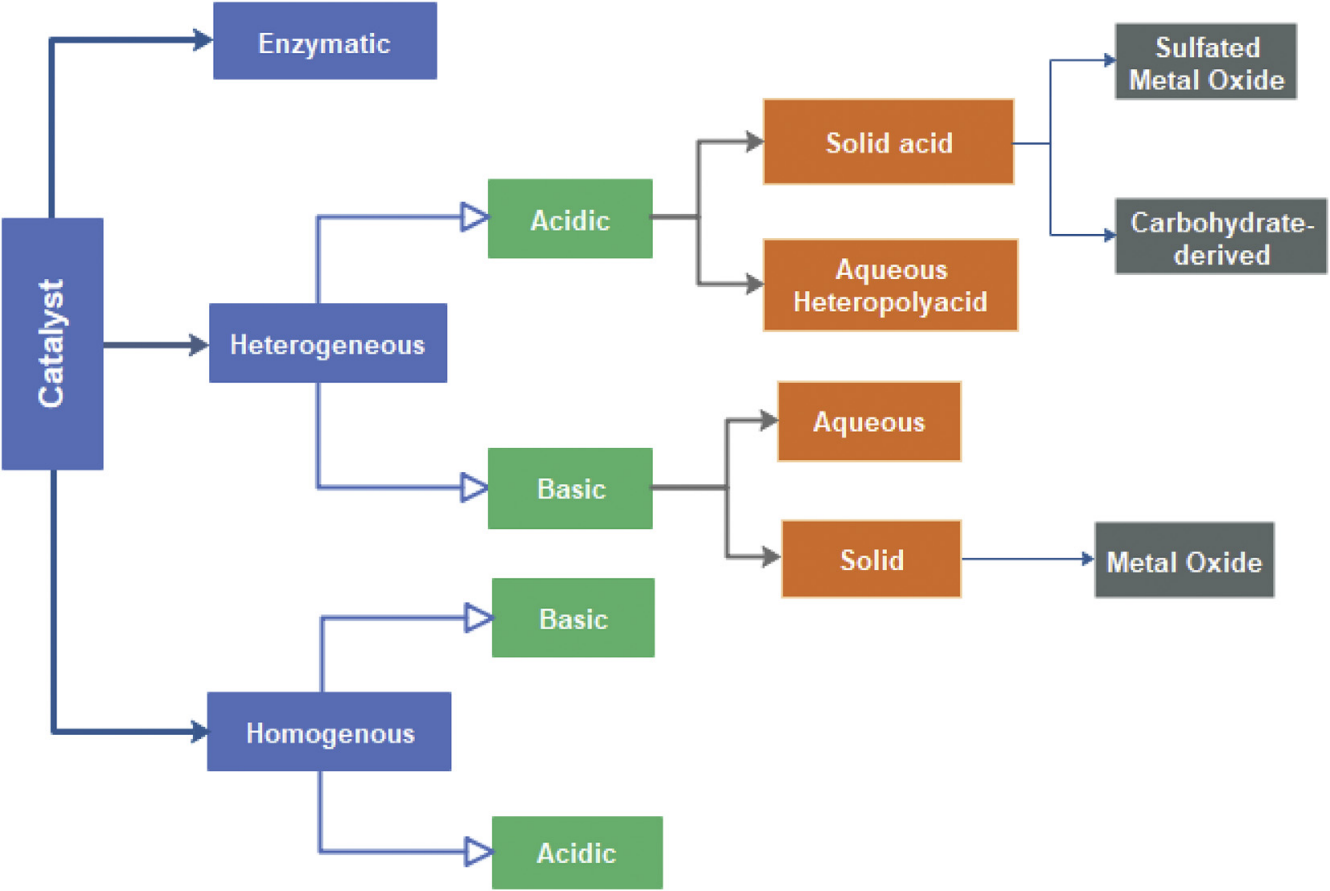
Overview of conventional catalyst types *(**Lokman* et al. *2015**)*.

Transesterification involves the conversion of the fatty acids chain of triglyceride molecules found in oil samples into ethyl or methyl esters in the presence of an alcohol and catalyst mixture (Folayan et al., 2019). The ASTM (American Society for Testing and Materials Standard) describes the mono-alkyl esters of fatty acids with long chains formed from the transesterification process as Biodiesel (Carlos et al., 2011; Ratanabuntha et al., 2018). Generally, the efficacy of converting oil to biodiesel through transesterification process depends on (i) reaction time, (ii) reaction temperature, (iii) ratio of alcohol to oil and (iv) the quantity of the catalyst (Abbah et al., 2016; Yusuff et al., 2019). Alcoholises occurs when alcohol is used for the transesterification process. This involves switching of organic “R” group of an ester with an “R” group of alcohol to produce glycerol and fatty acid alcohol ester (Ayhan, 2008). The catalyst mixture can be in the form of an alkaline, acidic or enzymes medium, respectively (Girish, 2018). Figure 2 illustrates the broad classifications of catalysts used in transesterification processes.

Potassium Hydroxide (KOH), Sodium Hydroxide (NaOH) and sulphuric acid (H_2_SO_4_) are the commonly selected soluble base catalyst for transesterification reactions (Yusuff et al., 2017; Zahan and Kano, 2018). The catalyst is mixed with methanol and stirred vivaciously in a reactor before being siphoned into the oil. Mixing continues until a decent transesterification reaction occurs with the appearance of two fluid layers: ester (biodiesel) and glycerol as a byproduct (Borugadda and Goud; 2012). Lam et al. (2019) applied boiler ash as a catalyst for transesterification of palm oil and successfully obtained a 94.48% yield. The reaction was conducted for 3 h using a 12:1 M ratio of methanol to oil and 15% by weight catalyst charge at 60 °C reaction temperature. The reactions’ high temperature/weight conditions reduce the probability of reusing the catalyst.

**Figure 3:**
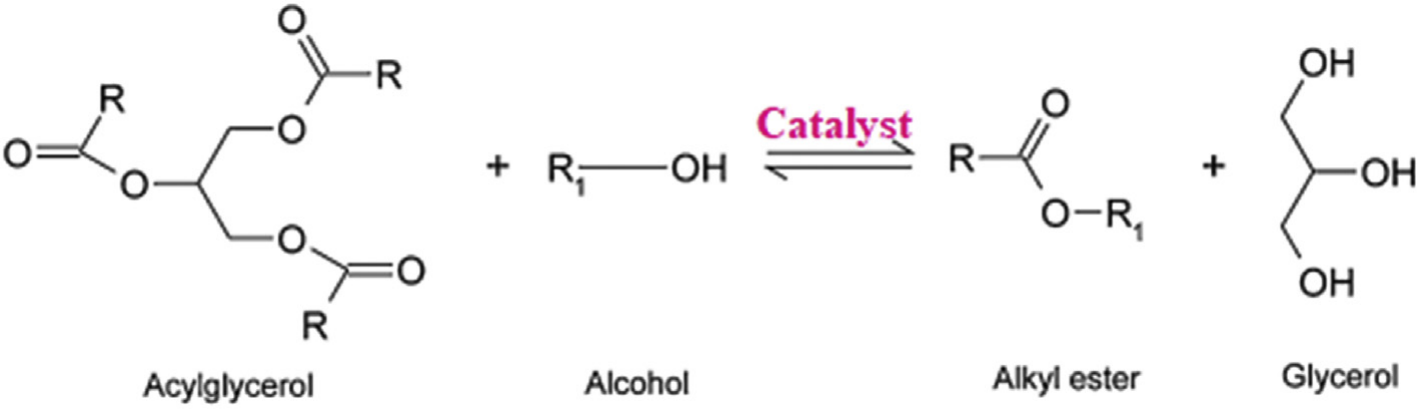
A typical transesterification reaction (Lourinho and Brito, 2015).

The major setback associated with the transesterification is unintended soap formation known as saponification. The saponification occurs as a by-product (Piloto-rodríguez et al., 2020) (Makgaba et al., 2018). Glycerol separation becomes difficult due to soap formation (Tabatabaei et al., 2015). Feedstocks with high fatty acid are first esterified to reduce fatty acid before the transesterification (Sharma et al., 2014). The cost of carrying out the esterification may be an extra cost allied with the production of biodiesel from some feedstock. Figure 3 shows a typical transesterification reaction structure. Monoglyceride and diglyceride are obtained from glycerol and alkyl ester reaction in subsequent stepwise reactions.

## 2 Methodology

### 2.1 Biodiesel production

The need to continually research to improve biodiesel production technologies as a solution to energy crises across Nigeria cannot be overemphasised. We experimentally assessed a locally available, clean and economical bioenergy source (i.e. Palm Olein) for biodiesel production for IC engines. The procedure followed is as below.

### 2.2 Transesterification procedure

**Figure 4:**
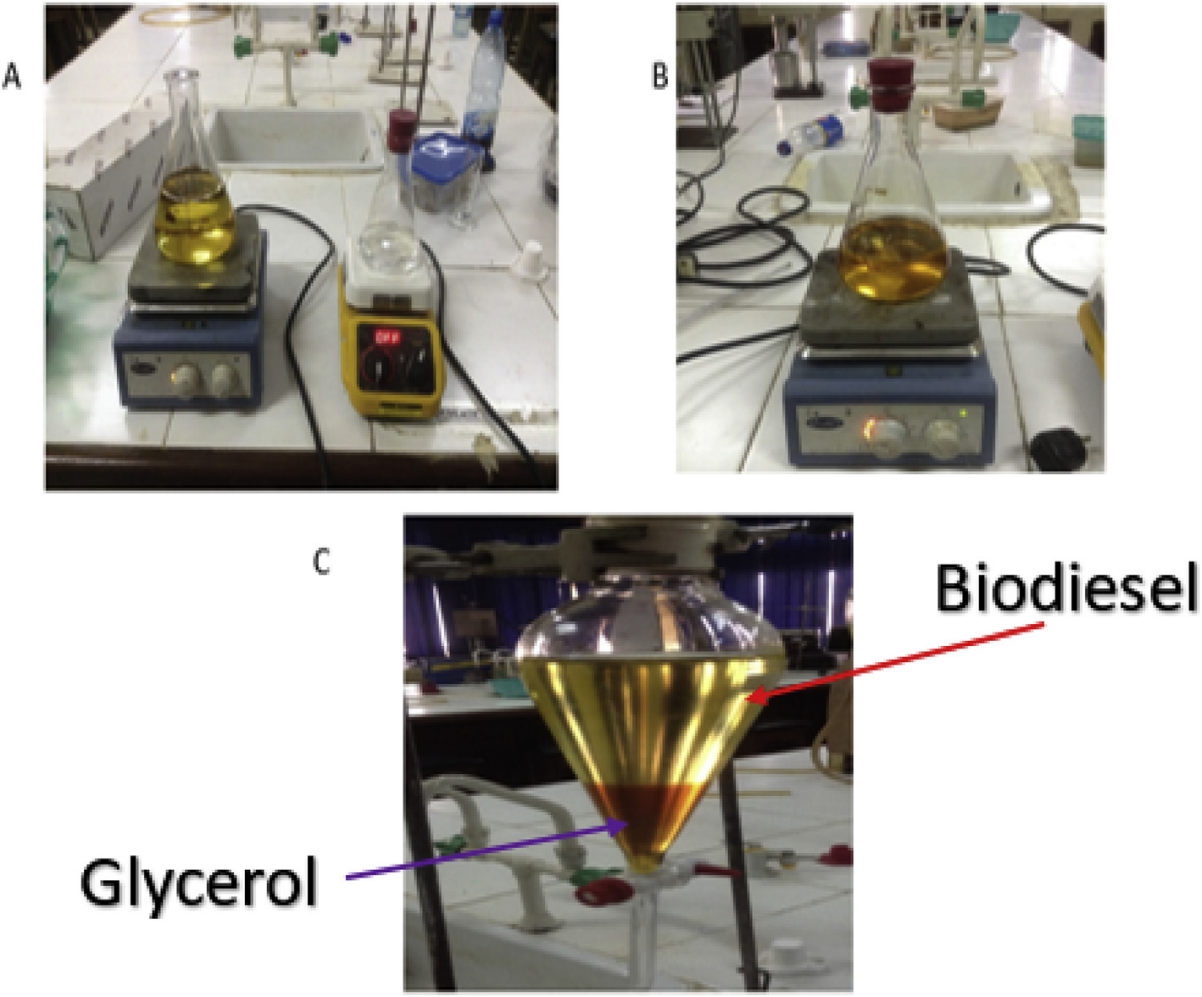
(A)-Oil Heating in Conical Flask and Mixture of Methanol and KOH (B)- The reaction of Oil with Methanol and KOH Mixture (C)- Separation of Product into Crude Biodiesel and Glycerol.

**Figure 5:**
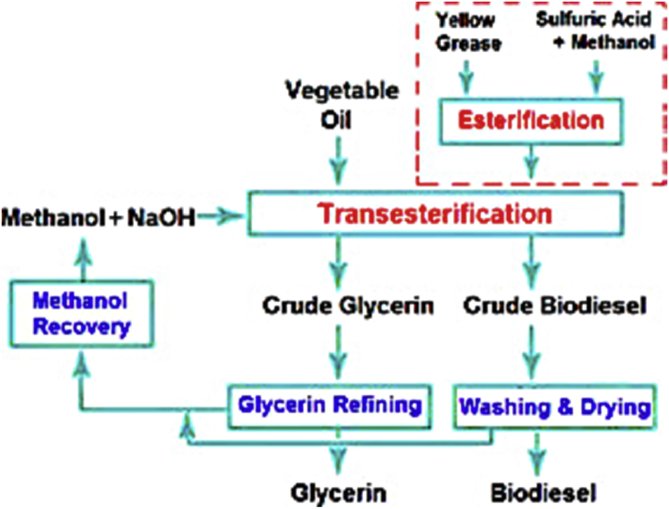
Schematic representation of transesterification process (Guo et al., 2015).

The RBD palm olein utilised for this experiment had an acid index of 10 mg KOH/g and was purchased from a local market in Ota, Nigeria. The acid index of the RBD was estimated following ASTM D974 recommendation (See Standard ASTM D 974–04). Trans-esterification method was adopted for the production of the respective biodiesels. About 200ml of palm olein oil was weighed into a conical flask, which was placed on a magnetic stirrer regulated to a temperature range of 60 °C and 65 °C. A methoxide solution containing 1.5g of KOH and 20% volume of methanol was added to the esterified oil. A reflux condenser was also set up to prevent the escape of the methoxide solution, while the reaction was stirred at five revolutions per minute (rpm) for 90 min. After a 24 h separation process, two distinct layers were formed, a darker coloured layer at the bottom (glycerol) and a layer of trans-esterified oil (biodiesel) at the top (See Figure 4c). The bottom layer was disposed of as waste. Figure 4 shows the experimental setup, while Figure 5 represents the schematic of the transesterification process.

### 2.3 Biodiesel washing and heating procedure

**Figure 6:**
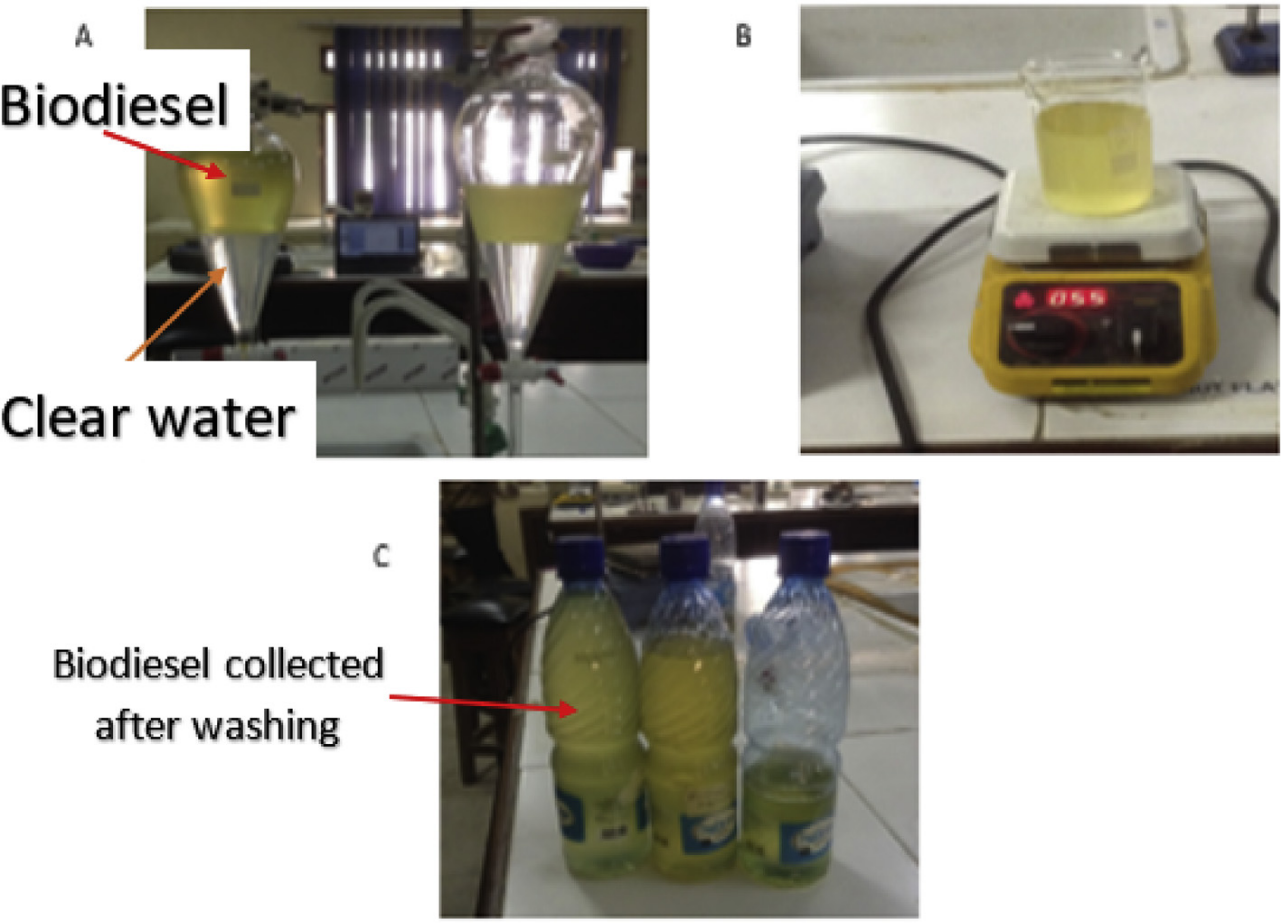
(a) Washing of Crude Biodiesel. (b) Drying of the Biodiesel. (c) Biodiesel obtained.

The biodiesel product has to be washed in other to remove every form of glycerine and impurities. The washing procedure involves boiled water added to the biodiesel and allowed to settle in a separating funnel for 12 h (see Figure 6a). The bottom layer was continuously removed until a transparent sample was obtained. The cleared biodiesel is emptied into a beaker and heated to 55 °C to remove water content (See Figure 6 a-c).

### 2.4 Measurement of parameters

#### 2.4.1 Density

The density of the biodiesel was determined using a specific gravity bottle. Firstly, the empty 50mL specific gravity bottle mass ($m_{1}$) was obtained using a digital weighing balance. Then the mass$\;(m_{2}$) of the specific gravity bottle filled to the brim with the biodiesel was recorded and adopted for the estimation of the biodiesel density using Eqs. (1) and (2).

(1) $$Density\left(kg/ml\right)=\frac{m₂-m₁}{v}$$

Where:

m₁ = mass of empty specific gravity bottle with the lid on it (kg)
m₂ = mass of specific gravity bottle filled with the oil (kg)
v = volumetric capacity of the bottle for specific gravity which is 50ml

(2) Density, kg/m^3^ = (specific gravity) x (997.6)

The density determination is based on standard ASTM D792 at 23 °C (Hariram et al., 2018).

#### 2.4.2 Flashpoint

A substance's flashpoint is defined as the lowest temperature at which the substance experiences ignition upon application of an ignition source. Biodiesel's flashpoint was determined using the closed cup flash point tester by ASTM D92 standard (Thushari and Babel, 2018).

#### 2.4.3 Viscosity

Viscosity is a measurement of the flow resistance of a fluid. The viscosity was measured to investigate the flow behaviour of the feedstock and biodiesel sample at 40 °C temperature. The viscosity tests were carried out using the OFITE automatic viscometer under standard ASTM D 445 (Saeed et al., 2019).

#### 2.4.4 Cetane rating

Cetane number is a rating of the quality of ignition and combustion of a fuel type. Cetane number is a dimensionless but the most influential fuel properties that are mostly responsible for the ignition delay as well as the ratio of premixed combustion to diffusion combustion in a diesel engine. It therefore widely affects the profile of heat release and also could be responsible for the emission of pollutants and combustion noise (Giakoumis and Sarakatsanis, 2019). The palm olein biodiesel was injected to fill up constant volume combustion closed cup of a handheld Labgent octane/cetane meter where it was compressed and ignited. The cetane was obtained by taking an average of results of 5 combustion curves. The method is by the ASTM D976 standard (Moser, 2009; Kaisan et al., 2017).

## 3 Results and discussion

### 3.1 Biodiesel production

Table 2 shows the parameters and result obtained from the three-run average data for the three batches of transesterification experiments process using methanol as alcohol.

**Table 2:** The Characteristics and Results of **t**hree-run average data for three selected batches of transesterification.

Experimental conditions	Case 1	Case 2	Case 3
KOH quantity (g)	0.75	1.5	3.0
Reaction temperature (°C)	65	65	65
Reaction time (minutes)	90	90	90
Palm olein quantity (ml)	100	200	400
Methanol quantity (ml)	20	40	80
Quantity of biodiesel obtained (ml)	73	150	295.5
Quantity of by-product obtained (ml)	45.5	90	180.5
Biodiesel yield (%)	61.6	62.5	62.1

Accuracy ±0.05 ml.

The calculation for Density of Fluids.

From Eq. (1);

given mass of palm olein oil in specific gravity bottle, m₂ = 78.0 g.

Mass of specific gravity bottle, m₁ = 31.9 g.

Volume of specific gravity bottle, v = 50 ml

$$Density\;of\;palm\;olein\;oil\;\left(g/ml\right)=\frac{\left(78.0-31.9\right)g}{50\;ml}=0.922\;g/ml$$

Mass of biodiesel in specific gravity bottle, m₂ = 76.1 g.

Mass of specific gravity bottle, m₁ = 31.9 g.

Volume of specific gravity bottle, v = 50ml

$$Density\;of\;biodiesel\;obtained\;\left(g/ml\right)=\frac{\left(76.1-31.9\right)g}{50\;ml}=0.884\;g/ml$$

*Density conversion to kg/m³*

1 g/ml = 1000 kg/m³

$$Density\;of\;palm\;olein\;oil\;\left(\frac{kg}{m^{3}}\right)=\;\left(\frac{0.922\;g/ml\;x\;1000\;kg/m³}{1\;g/ml}\right)=922\;kg/m³$$

$$Density\;of\;biodiesel\;obtained\;\left(\frac{kg}{m^{3}}\right)=\left(\frac{0.884g/ml\;x\;1000\;kg/m³}{1\;g/ml}\right)=884\;kg/m³$$

According to the ASTMS D792 (Hariram et al., 2018), Table 3 shows that palm olein based biodiesel falls within the acceptable standard specifications for diesel fuel. In Table 3, the developed biodiesel density was 8.47 % higher than the base diesel fuel. The experimental implication of infusing high-density fuel into IC engines is that more mass of fuel would be available for combustion within the system. Thus, improving the volumetric efficiency and power of the system. Besides, the cetane number of developed biodiesels reduced by 2.18 %. Although low cetane number of fuels are responsible for ignition delays, the reduction in the cetane number of the developed biodiesel is negligible. It cannot negatively impact on the performance of IC engines. This is because the cetane number of the biodiesel is about 4.06 % higher than the least required cetane number (i.e. 47) recommended by D792 ASTM standard (See Table 3). The flashpoint of the developed palm olein biodiesel is 208 °C, which is higher than pure diesel and D792 ASTM recommended fuels having 85 °C and 52 °C flash points respectively.

**Table 3:** Properties of biodiesel obtained in comparison to petroleum diesel and ASTM standard.

	Palm olein oil	Olein Biodiesel	Diesel	ASTM standard
Density	922 kg/m³	884 kg/m³	850 kg/m³	-
Flash point	323.9 °C	208 °C	85 °C	52 °C (min)
Cetane Number	-	48.91	50	47

### 3.2 The transesterification overall yield of the palm olein

For the optimum mix, 2.4 L of palm olein oil reacted with methanol to produce 1.5 L of biodiesel and 0.9 L of glycerol. The average transesterification trials gave 62.5% biodiesel yield from the oil. Table 4 shows the comparison between the critical properties of some other crop biodiesels researched in Nigeria by some authors. The table summarises the results of the characterisation performed on the produced biodiesel used and the results were compared to the attainable standard and the available literature. The fundamental properties considered include yield (%), specific gravity, kinematic viscosity, flash point, cetane index, iodine, heat and acid value, density, method of production, the catalyst used, the temperature and reaction duration. While almost all the presented biodiesel research outcomes presented conforms to ASTM standards, Coconut Oil has a flashpoint lower than the ASTM recommendation. It can be seen that the feedstock utilised in the production of biodiesel yield of this study is comparatively low. The 62.5% yield obtained in this study can be considered adequate.

**Table 4:** Comparison between the critical properties of some types of crop biodiesels available in Nigeria.

Parameters	Biodiesel Type										
	ASTM STANDARD	RBD Palm Olein	Palm Oil	Palm Kernel Oil	Coconut Oil	Avocado Seed Oil	Jatropha Oil	Soybean Oil	Groundnut Oil	Sweet Almond Oil	
*References*	(A.L. Paul Anawe and Adewale, 2018)	*Present Study*	(Kareem et al., 2017)	(Akhabue and Ogogo, 2018)	(Musa et al., 2016)	(Dagde, 2019)	(Eboibi et al., 2018)	(Ayodeji et al., 2018)	(Oniya and Bamgboye, 2014)	(Giwa and Ogunbona, 2014)	*Units*
Method		TE	TE	TE-Opt	TE	TE	TE	TE	TE	TE	
Yield		62.5	95.3	94.6	49.8	78	45	97.1	86.8	85.9	**%**
Density		0.884	0.813	0.88	0.89	0.86	0.92	0.87	0.85		*g/cm*^*3*^
Kinematic Viscosity	1.9–6.0	4.56	4.9	4.64	2.7	3.94	2.65	2.92	7.60	4.23	*mm*^*2*^*/s****at 40***^**°**^**C**
Flashpoint	130 (min)	208	270	162	100	162	146	140	200	169	^*o*^*C*
Cetane Number	47 (min)	48.91	N/A	N/A	51	62.2	N/A	41.36	N/A	58	
Iodine Value	N/A	N/A	N/A	72.8	124.6	38.2	N/A	N/A	0.21	92.3	*gl*_*2*_*/100g*
Heating Value	N/A	N/A	N/A	N/A	49	N/A	N/A	N/A	30.07	N/A	*mj/kg*
Acidic Value	0.80 (max)	N/A	N/A	0.62	0.18	0.89	N/A	N/A	N/A	0.13	*mg/KOH/g*
Catalyst/Temp. ^o^C/Reaction Time		KOH 65 °C (1.5)	Lipase 40 °C (0.5)	CaO 60 °C (2.25)	NaOH 65 °C (1.5)	KOH 65 °C (0.4)	NaOH 60 °C (1.5)	NaOH 60 °C (3)	KOH 60 °C (1)	NaOH 60 °C	*(Hrs)*

TE- Transesterification; Opt- Optimised; min – Minimum acceptable value; max - Maximum acceptable value.

N/A- Not available within the considered study.

### 3.3 The FTIR analysis

Fourier transforms infrared (FTIR) spectroscopy is one of the most commonly used spectroscopic techniques to identify compounds. Recommendable merits of this method include the high sensitivity, and it is a relatively inexpensive technique for carrying out such analysis (da Silva et al., 2017). Absorbance measurements in the infrared range were obtained by FTIR analysis using a Nicolet iS10 FT-IR Spectrometer model. The measurements were made with a resolution of 4cm^−1^, a transmission from 4000 to 350cm^−1^; performing 14 scans. The spectra were obtained at room temperature.

**Figure 7:**
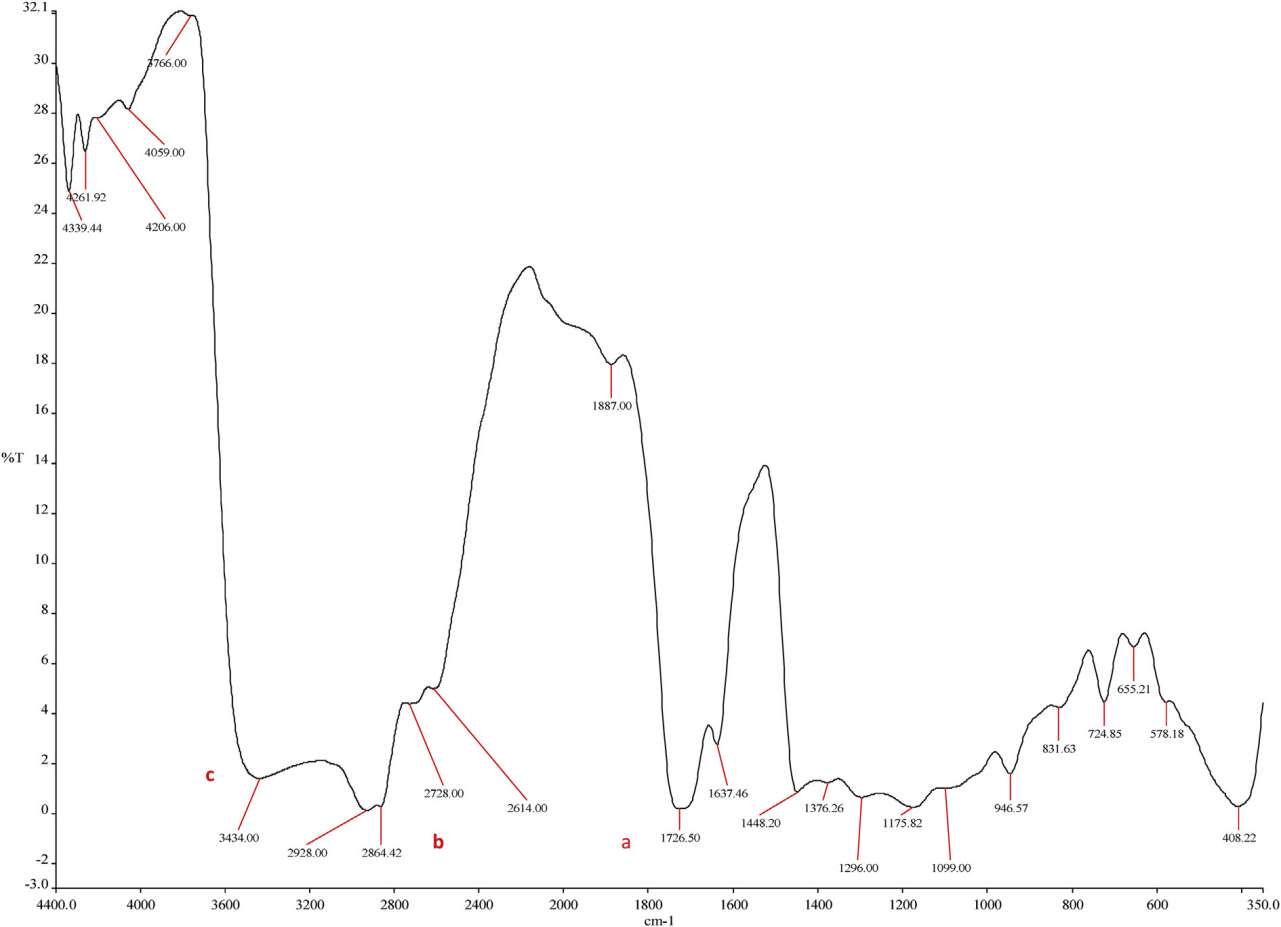
FTIR chart for the RBD Palm Olein Oil.

In this study, the FTIR spectra of the subfractions of the biodiesel components were recorded against attenuated total reflectance as a benchmark. During the sample analysis, absorption and transmission data versus wavelengths were logged and plotted, as shown in Figures 7 and 9. The spectral information represents the experimental data analysed by using principal component analysis charts.

**Figure 8:**
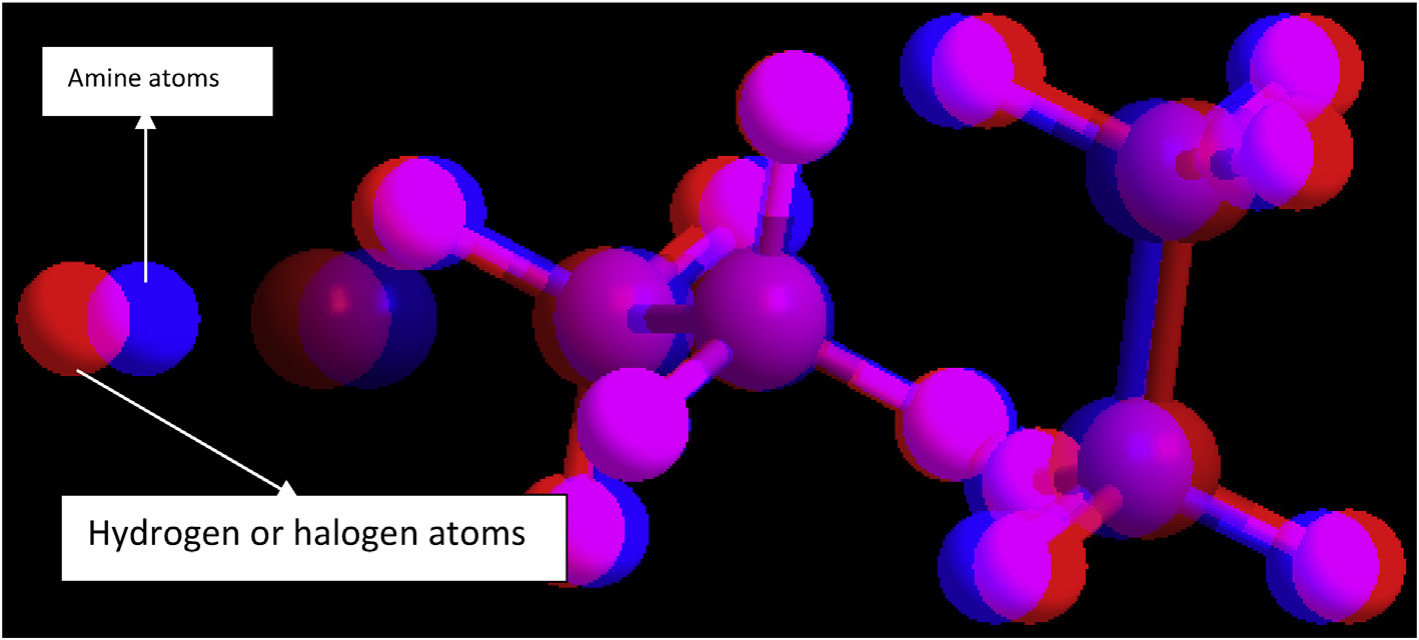
A simulated amine stretch compounds representation.

The FTIR analysis of the palm olein oil shown in Table 5 affirms that it is a carbon-rich compound. The strong reactions that promoted the favourable output were induced by the carboxylic acid (esters), the methyl stretch and amine stretch present in the oil as illustrated by a, b and c respectively in Figure 7. The RBD palm olein oil has about 31% residue from wavenumber 3766.00cm^−1^ to 4339.44cm^−1^ owing to their carbon origin, which are indications of the presence of high energy storage in the compound. The implementation of the amine stretch compounds is as simulated by ChemDraw software in Figure 8. The blue dot represents the Amine; the red dots illustrate its articulation with hydrogen or halogen atoms.

**Table 5:** FTIR Wavenumber interpretation for the RBD Palm Olein Oil.

Wavenumber (cm^−1^)	Intensity	Functional Group
408		Fingerprint region
655.21; 724.85	*M*	C–Cl stretch
946.57	M	O–H bend
1296.00	*M*	C–N stretch
1448.20	*s-w*	-CH3 aromatic
1637.46	*M*	C=C aromatic ring
1726.50	*S*	C=O stretch
1887.00	*W*	= C–H stretch
2614.00	*W*	-O-H Stretch
2728.00	*M*	C–H Stretch
2864.42	*m-s*	= -CH_3_ stretch
2928.00	*M*	-C-H stretch
3434.00	*S,b*	N–H stretch
3766.00		Residue
4059.00		Residue
4206.00		Residue
4261.92		Residue
4339.44		Residue

*s*-strong, *b*-broad, *m*-medium, *w*-weak, *n*-narrow.

**Figure 9:**
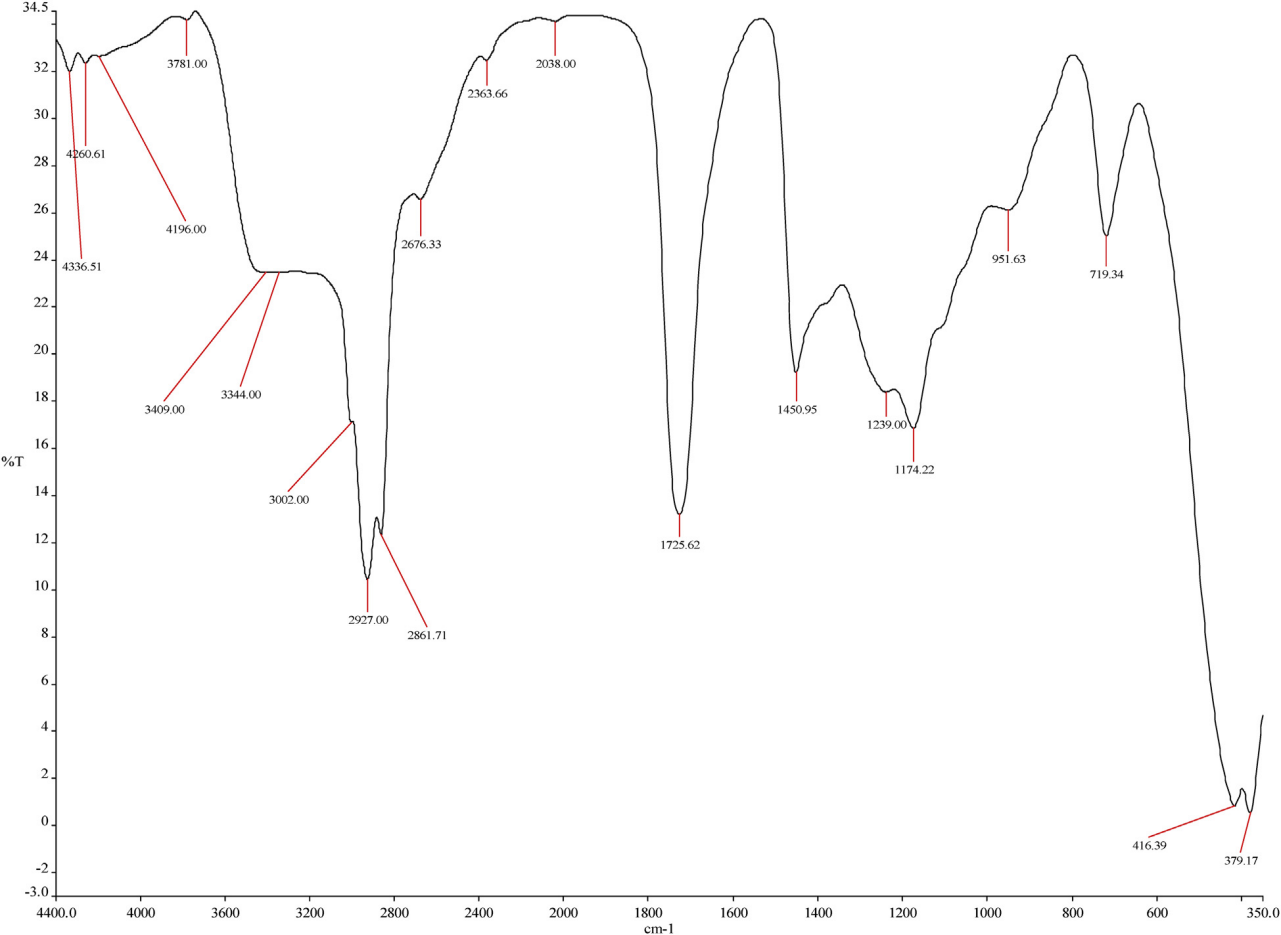
FTIR chart for the Palm Olein Biodiesel.

The FTIR analysis representations (Figure 7 and Figure 9) have minor differences between their spectra because the product (biodiesel) of the transesterified palm olein is chemically similar to its precursor (palm olein oil). The two samples followed transmission peaks of the order ranging between 3300cm^−1^ and 2500 cm^−1^, which indicates the presence of OH in the free carboxylic acids. This suggests that fatty acids are present in the two samples in the form of carboxylic acids with an R−(C=O)–OH structure (da Silva et al., 2017). This is rightly so as the molecules in biodiesels are primarily fatty acid methyl esters (FAME) which is predominantly present in the feedstock as free fatty acids and triglycerides (Boey et al., 2011) (Kolhe et al., 2017) (Akinfalabi et al., 2019). For both spectra, characteristic peaks can be observed in the ranges of 3200cm^−1^ to 2800cm^−1,^ which are due to the = C–H stretching suggesting alkenes (Daniyan et al., 2019). Alkanes and alkenes are essential constituents of fuels for a wide range of industrial applications and the same for biofuels. Also for both spectra; infrared absorption between 1800cm^−1^ to 1700cm^−1^ (from Figure 7; strong intensity absorption at 1726.50 cm^−1^ for the oil) indicated C=O stretching typical for esters carbonyl compounds (Adeyinka S Yusuff et al., 2019). Also, similar peaks were noticed between 1600cm^−1^ and 1000cm^−1^ for both the feedstock oil and the product biodiesel, which are due to CC ring connoting presence of esters and ethers (Mohamed et al., 2018).

The peak at 1448.2cm^−1^ corresponds to the asymmetric stretch of -CH3 aromatic which can be attributed to the glycerol groups present in the spectrum of RBD palm olein oil and noticeably missing in the spectrum for the biodiesel, as the result of the separation of glycerol from the extracted biodiesel (Rabelo et al., 2015). Another region in which the biodiesel and the RBD oil can be distinguished is 1300cm^−1^ to 350cm^−1^, known as the "fingerprint" region. The spectral range confirms the identity in connections with absorptions resulting from the stretching of the CO bond of the esters; thus, corroborating the earlier observation (Mueller et al., 2013). Comparing the two FTIR analysis, the fingerprint region is more prominent (strong absorption intensity) in the spectrum of RBD palm olein oil than the spectrum of biodiesel confirming the formation of energy-rich mono alkylate product synonymous to fuels (Hiwot, 2017).

The FTIR spectrograph shows the different functional groups present in this composite in Table 6. The absorbance at 1174.22cm^−1^ (Figure 9) is typical of biodiesel; it represented the C–O stretch in the form of O–CH_3_. According to Domingos et al. (2017); the bands less than 1244cm^−1^ are due to the asymmetric axial vibrations of the saturated aliphatic esters, and the bands between 1169 and 1196cm^−1^ are caused by axially asymmetric deformation groups of the aliphatic esters.

**Table 6:** FTIR Wavenumber highlight for the RBD Palm Olein Biodiesel.

Wavenumber (cm^−1^)	Intensity	Functional Group
379.17; 416.39; 719.34; 951.63		Fingerprint region
1174.22	*M*	C–O stretch
1450.95	s-w	Aromatic
1725.62	*n,s*	C=O stretch
2363.66	*W*	C≡N Stretch
2676.33	*W*	O–H stretch
2861.71	*W*	-C-H stretch
2927.00	*M*	C–H Stretch
3002.00	*W*	C–H Stretch
3344.00	*m,b*	N–H stretch
3409.00	*b,w+*	O–H stretch
3781.00		Residue
4196.00		Residue
4260.61		Residue
4336.51		Residue

*s*-strong, *b*-broad, *m*-medium, *w*-weak, *n*-narrow.

**Figure 10:**
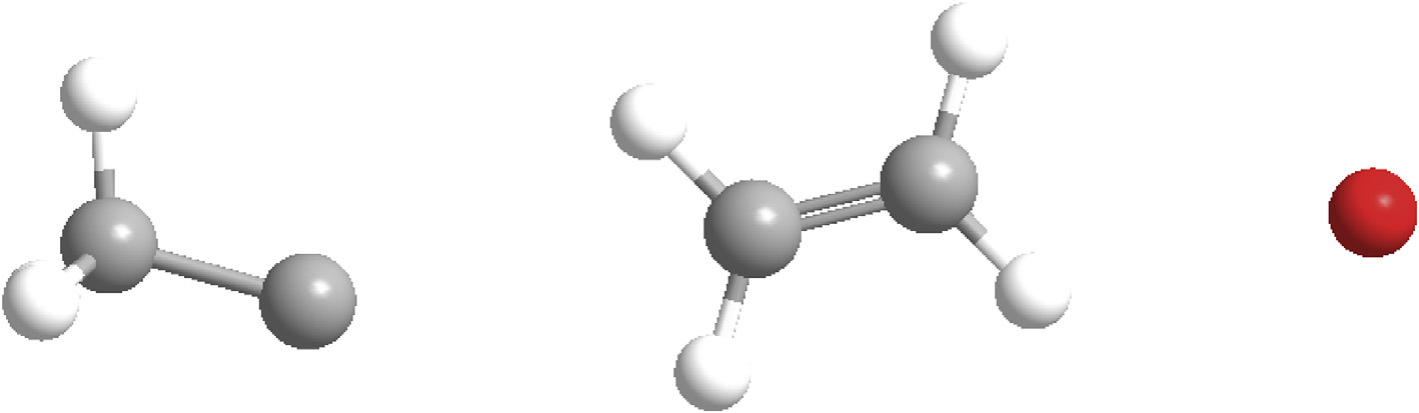
A prototypical representation of carboxylic groups.

The aromatic and carboxylic stretch (3600-2700 cm^−1^) gives some weak, medium and broad reactions (in Figure 9) which indicated fuel product pattern. The significance of which is simulated with ChemDraw, as shown in Figure 10. In comparison to the feedstock (palm olein), the biodiesel has less residue, which could be as a result of the transesterification and the purification process. Another pertinent factor is the intensity of Amine in the feedstock, which does promote the desired output in transesterification. However, the biodiesel favoured a higher degree of the production of esters, which is the desired product.

## 4 Conclusion

This research ascertained the feasibility of producing biodiesel from a Nigerian sourced palm olein with the use of batch reactors. The percentage yield of biodiesel using methanol in the presence of Potassium hydroxide (KOH) catalyst was 62.5%, thus confirms its feasibility for mass production. The biodiesel derived has a density of 0.884 g/cm^3^, cetane number of 48.91 and a flashpoint of 208 °C, in tandem with the values based on ASTM D975 Standard Specification for diesel fuels. This investigation established that the RBD palm olein is suitable for commercial-scale biodiesel production. The Nigerian government and stakeholders are encouraged to utilise the bio-resources capability of the oil palm industry via palm olein based biodiesel. However, further investigation is recommended for optimisation of catalyst type and concentration, reaction time and temperature, and the mole ratio of the reagents; to improve the quality of the biodiesel as well as increase the yield.

## Declarations

### Author contribution statement

Felix Ishola: Conceived and designed the experiments; Wrote the paper.

Damola Adelekan: Conceived and designed the experiments; Performed the experiments; Wrote the paper.

Angela Mamudu: Performed the experiments.

Temitope Abodunrin & Abraham Aworinde: Analyzed and interpreted the data.

Obafemi Olatunji & Stephen Akinlabi: Contributed reagents, materials, analysis tools or data.

### Funding statement

The authors acknowledge Covenant University, Ota, Nigeria for funding the publication.

### Competing interest statement

The authors declare no conflict of interest.

### Additional information

No additional information is available for this paper.
